# Influence of Quenching and Subsequent Annealing on the Conductivity and Electromechanical Properties of Na_1/2_Bi_1/2_TiO_3_-BaTiO_3_

**DOI:** 10.3390/ma14092149

**Published:** 2021-04-23

**Authors:** Lalitha Kodumudi Venkataraman

**Affiliations:** Department of Materials and Earth Sciences, Technical University of Darmstadt, 64283 Darmstadt, Germany; venkataraman@ceramics.tu-darmstadt.de

**Keywords:** lead-free piezoceramics, quenching, Na_1/2_Bi_1/2_TiO_3_, oxygen vacancies, thermal depolarization

## Abstract

Na_1/2_Bi_1/2_TiO_3_-based materials have gained considerable attention for their potential to exhibit giant strain, very-high ionic conductivity comparable to yttria stabilized zirconia or high mechanical quality factor for use in high power ultrasonics. In recent times, quenching Na_1/2_Bi_1/2_TiO_3_-based compositions have been demonstrated to enhance the thermal depolarization temperature, thus increasing the operational temperature limit of these materials in application. This work investigates the role of quenching-induced changes in the defect chemistry on the dielectric, ferroelectric and piezoelectric properties of quenched Na_1/2_Bi_1/2_TiO_3_-BaTiO_3_. The quenched samples indeed demonstrate an increase in the bulk conductivity. Nevertheless, while subsequent annealing of the quenched samples in air/oxygen atmosphere reverts back the depolarization behaviour to that of a furnace cooled specimen, the bulk conductivity remains majorly unaltered. This implies a weak correlation between the defect chemistry and enhanced thermal stability of the piezoelectric properties and hints towards other mechanisms at play. The minor role of oxygen vacancies is further reinforced by the negligible (10–15%) changes in the mechanical quality factor and hysteresis loss.

## 1. Introduction

The impending regulation that revived the interest on lead-free piezoelectric alternatives [1] identified four major families of materials based on BaTiO_3_ [2], Na_1/2_Bi_1/2_TiO_3_ [3], K_0.5_Na_0.5_NbO_3_ [4] and BiFeO_3_ [5,6]. Amongst these, Na_1/2_Bi_1/2_TiO_3_-based compositions are versatile and when appropriately modified, demonstrate potential use in (a) solid oxide fuel cells (high ionic conductivity) [7] and (b) high temperature capacitors [8] and high-power ultrasonics [9,10] (insulating character with high mechanical quality factor). In particular, Na_1/2_Bi_1/2_TiO_3_-based materials exhibit superior mechanical [11] and high power [9] properties in comparison to the lead-based Pb(Zr,Ti)O_3_. Further, processing strategies such as chemical doping [12,13,14], composite formation [15,16,17] and quenching [18] have been demonstrated to significantly alter the conductivity [19], strain [20], mechanical quality factor [14,21,22] and depolarization temperature [14,16,23]. In recent years, quenching from sintering temperature has been adopted as a route to enhance the depolarization temperature (T_d_) of Na_1/2_Bi_1/2_TiO_3_ (NBT) [18]. Quenching was also shown to be effective in tailoring the ergodicity of NBT-based materials [24].

Na_1/2_Bi_1/2_TiO_3_-BaTiO_3_ (NBT-BT) solid solution exhibits a morphotropic phase boundary (MPB) which spans a wide range of compositions from 5–11 mole% BT [25,26] with varying degrees of average structural distortions (rhombohedral to pseudocubic to tetragonal with increasing BT content). The exact range of the MPB is also often debated depending on the synthesis routes [27] and control of stoichiometry [28]. The compositions at the MPB of NBT-BT are non-ergodic relaxors, which undergo an irreversible transformation from the relaxor to ferroelectric state upon application of stress [29] or electric field [25]. Upon heating the stress/field induced ferroelectric state, it transforms back to the relaxor state at the ferroelectric-relaxor transformation temperature (T_F-R_), which sets the upper limit for the depolarization temperature. T_F-R_ is typically established from the first anomaly in the poled temperature-dependent dielectric response [30]. Quenching has been effective in enhancing the T_d_ of NBT-BT, with significant changes (increase by 40–60 °C) noted for the non-ergodic relaxor compositions [23]. Based on the annealing study in oxygen atmosphere, quenching-induced increase in oxygen vacancy concentration was proposed to be the mechanism of enhanced T_d_ [23]. The quenched samples, subsequently annealed in oxygen atmosphere at 800 °C for 12 h, exhibited a reversal of T_F-R_ back to that of the furnace cooled state. Quenching disrupts the equilibrium concentration of oxygen vacancies at room temperature due to frozen-in defects from the high temperature state. The reversal of T_F-R_ upon annealing the quenched sample would then imply that annealing should also affect the defect population and enable equilibration of defects. Additionally, the domain wall mobility in ferroelectrics is greatly influenced by point defects such as oxygen vacancies, that as mobile/coupled defect complexes constrain the domain wall motion, resulting in hardening of the electromechanical properties [31,32]. Therefore, it is imperative to investigate the effect of the increased defect concentration on the electrical properties. Specifically, it is of interest to investigate the influence of quenching on the mechanical quality factor, which is one of the important figures of merit for high power applications, such as in ultrasonics. This work aims to establish the influence of quenching and subsequent annealing of Na_1/2_Bi_1/2_TiO_3_-BaTiO_3_ on the ferroelectric hysteresis and mechanical quality factor to gauge the hardening effects and rationalize the results based on the electrical conductivity.

## 2. Materials and Methods 

(1−*x*)Na_1/2_Bi_1/2_TiO_3_-*x*BaTiO_3_ (NBT100*x*BT) (*x* = 0.06, 0.09; *x* denotes number of moles) ceramics were prepared using the conventional solid-state reaction route. Stoichiometric ratios of Na_2_CO_3_ (99.5%), BaCO_3_ (99.8%), Bi_2_O_3_ (99.975%) and TiO_2_ (99.6%) (all Alfa Aesar, Thermo Fisher Scientific GmbH, Kandel, Germany) were milled in ethanol at 250 rpm for 24 h. The purity of the starting raw materials are indicated in brackets. The powders were calcined at 900 °C for 3 h with a heating rate of 5 °C /min. After calcination, all the powders were remilled in ethanol at 250 rpm for 24 h. The remilled powders were then cold isostatically compacted at 350 MPa into disks. To prevent volatilization of sodium and bismuth, the compacted green bodies were embedded in a powder bed of the same composition. Subsequently, following a heating rate of 5 °C/min, sintering was performed at 1150 °C for 3 h. These samples were removed from the furnace after they cooled to room temperature and are denoted as “FC” (furnace cooled). The samples that were directly taken out from the furnace after the sintering dwell time and cooled in ambient air using a fan are denoted as “Q1150” (quenched). The samples were ground to remove the surface. Next, annealing was done at 400 °C for 30 min to relieve the mechanically induced stresses that resulted from the grinding step. The final dimensions of the samples were 7.6–7.7 mm in diameter and 0.6 mm thick. The samples were then sputtered with Pt to obtain electroded surfaces. Poling was done at room temperature under an electric field of 6 kV/mm for 15 min. Permittivity measurements were carried out using a HP analyzer interfaced with a furnace in the temperature regime from ambient temperature to 500 °C with a heating rate of 2 °C/min. Polarization- and strain- electric field hysteresis loops were recorded with a triangular field at a frequency of 1 Hz using a Sawyer-Tower circuit coupled with an optical sensor. Resonance measurements were performed on poled samples using an impedance analyzer (Alpha-Analyzer, Novocontrol, Montabaur, Germany) and the mechanical quality factor was determined using the 3 dB method [33]. The electrical conductivity was measured using the same impedance analyzer. The impedance dataset was evaluated with the help of RelaxIS (rhd instruments, Darmstadt, Germany). Annealing experiments in air and oxygen atmosphere were performed at 800 °C for 12 h in accordance with the prior study [23] and the impedance spectra was measured in situ during the annealing step. All the other electrical characterization (temperature-dependent permittivity, hysteresis and mechanical quality factor) was done ex situ by measuring the response before and after the annealing step. Quenched samples annealed in air and oxygen atmosphere are denoted as “Q-Air800” and “Q-O_2_800” respectively.

## 3. Results and Discussion

### 3.1. Influence of Annealing on the Dielectric Properties of Quenched Samples

**Figure 1:**
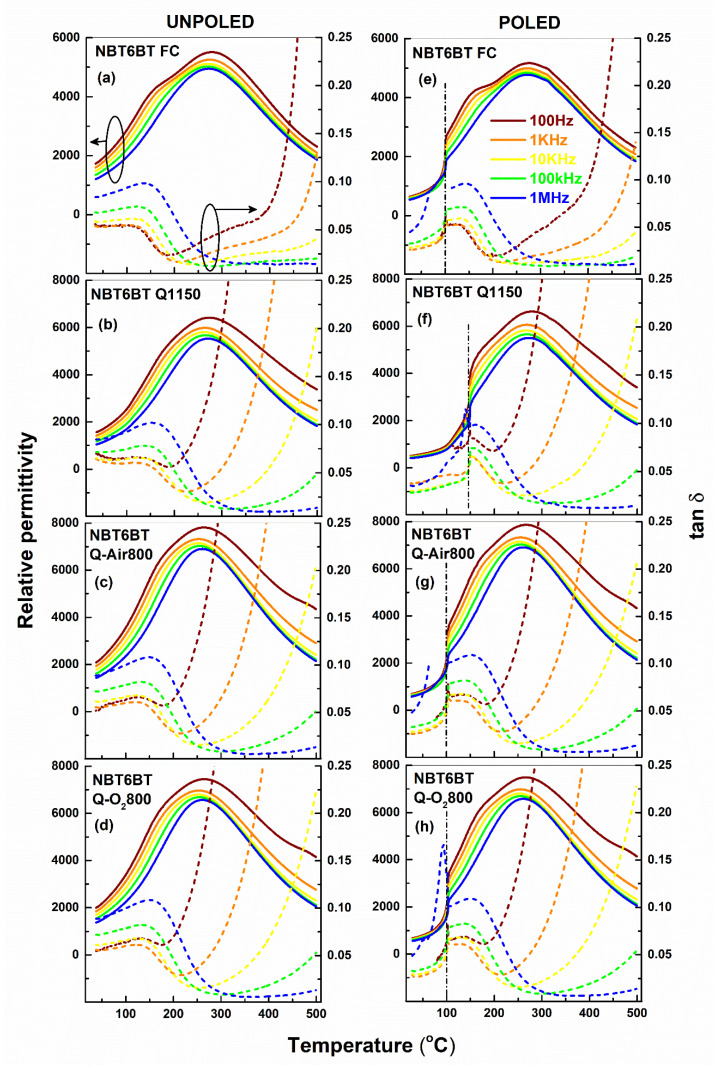
Temperature-dependent permittivity of NBT6BT for the (**a**,**e**) furnace cooled, (**b**,**f**) quenched and (**c**,**d**,**g**,**h**) quenched samples annealed in air/oxygen atmosphere in the unpoled and poled state. The vertical lines (dash-dot) in the poled dataset correspond to T_F-R_.

**Figure 2:**
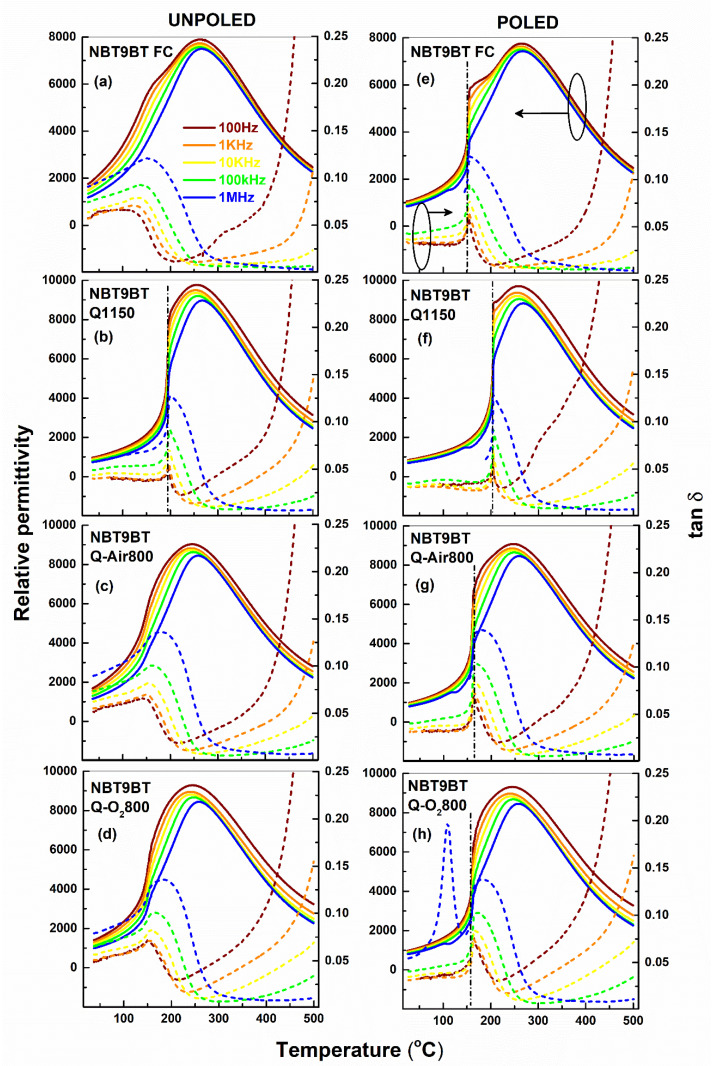
Temperature-dependent permittivity of NBT9BT for the (**a**,**e**) furnace cooled, (**b**,**f**) quenched and (**c**,**d**,**g**,**h**) quenched samples annealed in air/oxygen atmosphere in the unpoled and poled state. The vertical lines (dash-dot) correspond to T_F-R_.

Figure 1 and Figure 2 depict the temperature-dependent permittivity response of NBT6BT and NBT9BT subject to different thermal treatments. In the unpoled state, NBT6BT and NBT9BT exhibit typical non-ergodic relaxor characteristics, exemplified by the frequency dispersion below the maximum in permittivity (Figure 1a and Figure 2a). While quenching (Figure 1b) and subsequent annealing (Figure 1c,d) of NBT6BT retains the characteristic frequency dispersion in the permittivity response (Figure 1b–d), the spontaneous ferroelectric transition in NBT9BT Q1150 results in a sharp anomaly even in the unpoled state (Figure 2b), akin to previous reports [23]. As noted previously [23], this anomaly disappears upon annealing the quenched sample in oxygen atmosphere (Figure 2d). Notably, annealing the quenched sample in air results in reversal of the spontaneous ferroelectric order in NBT9BT Q1150 (Figure 2c), akin to the oxygen annealing study done in prior work [23]. The vertical dash-dotted line in the poled permittivity plots denote T_F-R_ (Figure 1e–h and Figure 2e–h, Table 1). NBT6BT FC and NBT9BT FC exhibit a T_F-R_ of 100 and 151 °C, respectively (Figure 1e and Figure 2e, Table 1). Albeit differences in the quenching strategy adopted in this study (directly from the sintering temperature, 1150 °C) compared to prior work (that involved cooling to 1100 °C and then quenching) [23], the increase in T_F-R_ upon quenching is comparable at 143 °C and 203 °C for NBT6BT Q1150 and NBT9BT Q1150 (Figure 1f and Figure 2f, Table 1), respectively. The dielectric loss (tan δ) at room temperature is comparable for the samples subject to different thermal treatments. The difference in permittivity at low and high frequency (Δε_Hz_) is used to establish the relaxor character based on the frequency dispersion, while the difference in permittivity between the unpoled and poled state (Δε_p_) can indicate the propensity to stabilize a ferroelectric order. Δε_p_ is lower for the quenched materials, indicating propensity to stabilize the ferroelectric order [23]. Δε_Hz_ is also lower for the quenched samples, although with marginal changes for NBT6BT Q1150, in comparison to the furnace cooled state. Upon annealing, both Δε_Hz_ and Δε_p_ increases than that of quenched samples and are comparable to that of the furnace cooled state. This is in accordance with previous reports, wherein annealing at temperatures above 800 °C was shown to revert the quenching induced changes in T_F-R_ to that of the furnace cooled state [23,24,25]. Note that the choice of annealing atmosphere (air or oxygen) does not significantly influence Δε_Hz_, Δε_p_ and T_F-R_ (Table 1). Δε_Hz_ and Δε_p_ for quenched NBT6BT subjected to annealing in air and oxygen atmosphere differ by < 4%. Δε_Hz_ of NBT9BT Q-Air800 and NBT9BT Q-O_2_800 differ by 24% (while Δε_p_ is similar), which is currently not understood. This could be related to the peculiar nature of compositions at the border of the MPB of NBT-BT phase diagram, which exhibit average non-cubic distortions and demonstrate ease of developing spontaneous ferroelectric order and reversal by changes in the thermal and poling history [34] or defect chemistry [35].

**Figure 3:**
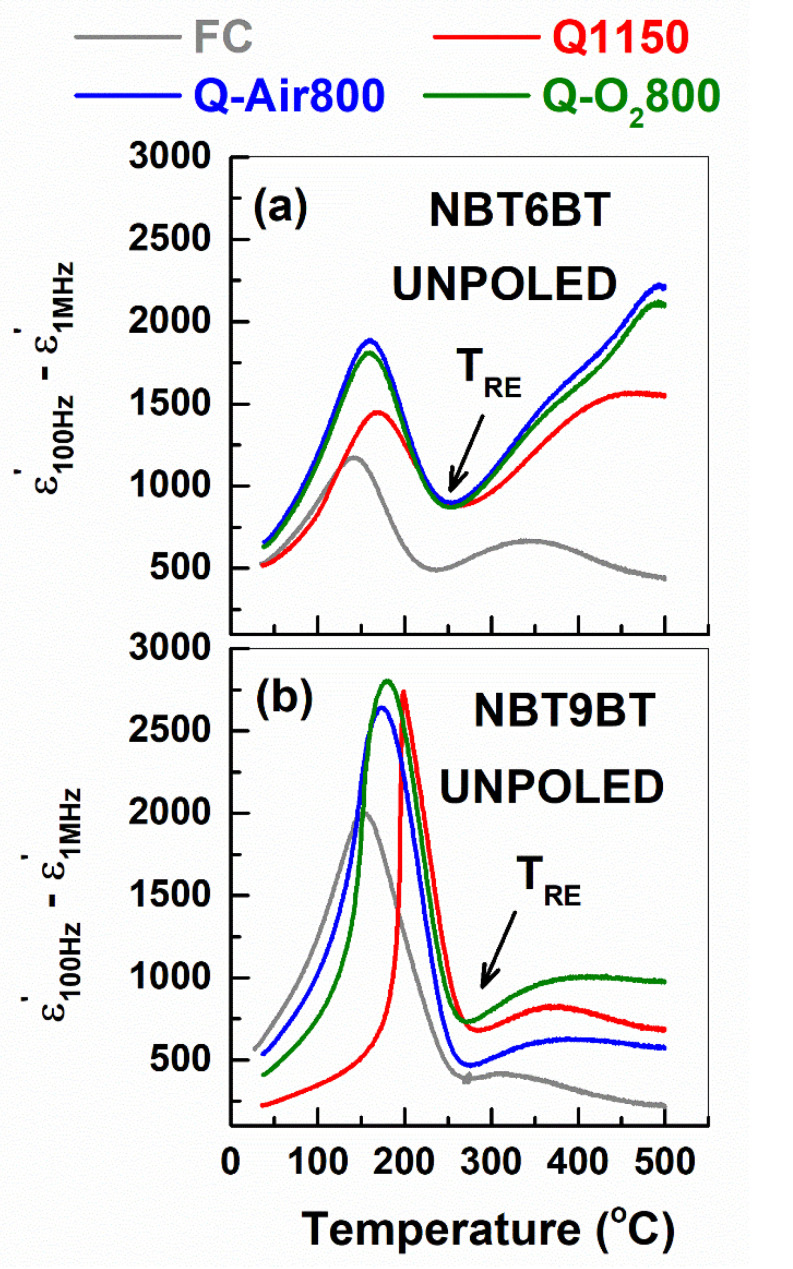
Temperature dependence of Δε_Hz_, given by $\varepsilon_{100Hz}^{’}\;$ −$\;\varepsilon_{1MHz}^{’}$ of (**a**) NBT6BT and (**b**) NBT9BT in the unpoled state for the furnace cooled, quenched and quenched samples subject to annealing in air/oxygen atmosphere. The solid arrows correspond to the upper limit of the frequency-dependent dielectric response (T_RE_).

The temperature corresponding to the maximum in permittivity (T_m_ in Table 1) is comparable for FC and Q1150 samples, as also noted for quenched NBT-BT-K_0.5_Na_0.5_NbO_3_ [24]. However, upon annealing, T_m_ decreases by 8–11 °C. The temperature dependence of Δε_Hz_ is plotted in Figure 3, wherein the minimum (indicated by solid arrows) corresponds to the upper temperature limit (T_RE_) of the frequency-dependent dielectric behavior [36]. Δε_Hz_(T) exhibits broader peaks for NBT6BT materials indicating strong frequency dispersion, while it is relatively narrow for NBT9BT materials. Δε_Hz_(T) of NBT9BT Q1150 exhibits a sharp peak, corresponding to the spontaneous ferroelectric transition [23]. T_RE_ is lower than T_m_ for NBT6BT FC, akin to prior reports [36] on relaxor NBT100*x*BT compositions. However, T_RE_ and T_m_ of NBT9BT FC are comparable (Table 1). For Q1150, Q-Air800 and Q-O_2_800 samples, T_RE_ < T_m_ for NBT6BT, while T_RE_ > T_m_ for NBT9BT. The permittivity is influenced by the changes in domain population and the number density of interfaces, for example domain or grain boundaries. While NBT6BT FC is characterized by weak lamellar domain contrast coexisting with polar nanoregions, NBT9BT FC exhibits a strong lamellar domain contrast at room temperature [37]. Further, quenching alters the phase assemblage and lamellar/PNR domain contrast in NBT100xBT, especially for the MPB compositions [37]. The difference in T_RE_ for the quenched and subsequently annealed samples of NBT6BT and NBT9BT can be rationalized by correlating to temperature-dependent changes of the domain morphology. However, it is beyond the scope of this study.

### 3.2. Annealing Effects on the Conductivity of Quenched Samples

**Figure 4:**
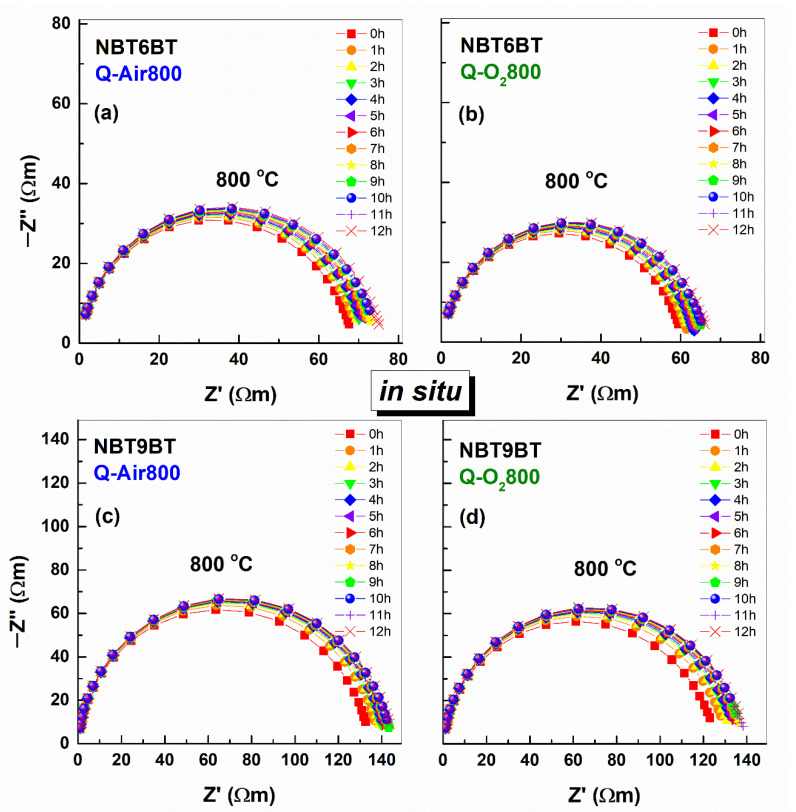
Nyquist plots of impedance measured in situ during annealing of the quenched NBT6BT and NBT9BT samples in (**a**,**c**) air and (**b**,**d**) oxygen atmosphere at 800 °C for 12 h.

**Figure 5:**
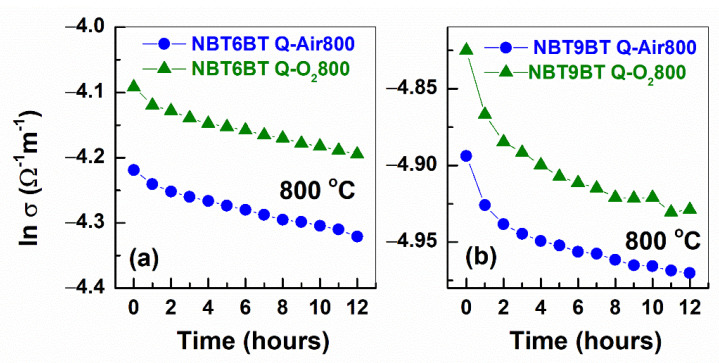
Conductivity as a function of time for quenched (**a**) NBT6BT and (**b**) NBT9BT samples annealed in air and oxygen atmosphere at 800 °C.

One of the hypotheses proposed earlier for enhanced T_F-R_ of quenched samples is the increase in oxygen vacancy concentration [23,38]. This perspective was further strengthened, when annealing in air/oxygen atmosphere was shown to revert the quenching induced changes in T_F-R_ back to that of the furnace cooled state [23,38,39]. Therefore, it is expected that annealing also alters the electrical conductivity of the quenched samples. Figure 4 depicts the Nyquist plots of impedance of quenched NBT6BT and NBT9BT at 800 °C as a function of time during in situ annealing in air and oxygen atmosphere. The plots feature a single semicircle for all the samples, indicating a single dominant conduction process. Further, the Nyquist plots are not very different as a function of annealing time. The dc conductivity evaluated from the low frequency response of the impedance dataset is plotted in Figure 5. Note that the conductivity of the quenched samples exhibit marginal changes as a function of time during the annealing step. In contrast, anneaing the the quenched NBT6BT samples in air at 800 °C for 2 h already decreases the T_F-R_ from 136 °C to 116 °C [38].

In the extreme case, annealing at 800 °C for 12 h reverts and decreases the T_F-R_ of the quenched state back to that of furnace cooled specimen (Table 1). The impedance analysis combined with the in situ annealing study and dielectric investigations (Section 3.1) indicate that the changes in T_F-R_ of the quenched and subsequently annealed samples are not accompanied by the changes in the conductivity. Therefore, these results establish weak correlation between the quenching-induced stabilization of ferroelectric order and the related changes in the defect chemistry. The negligible influence of the annealing atmosphere (air/oxygen) holds true here as well, exemplified by the similar bulk conductivities (<3% difference) of air and oxygen annealed samples (Figure 5).

**Figure 6:**
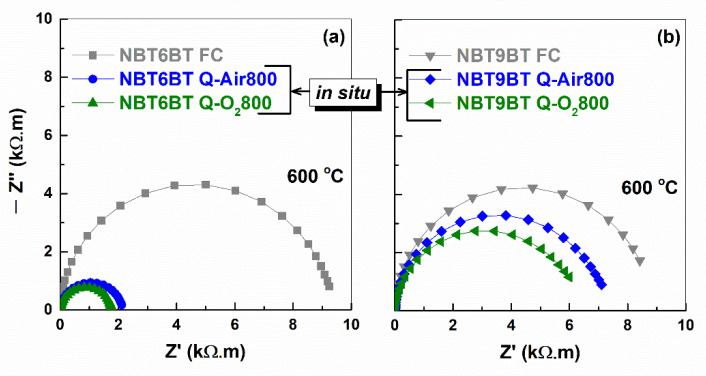
Nyquist plots of impedance for furnace cooled and quenched (**a**) NBT6BT and (**b**) NBT9BT samples at 600 °C. The plots for the quenched samples correspond to the heating step at 600 °C during in situ annealing in air and oxygen atmosphere.

**Figure 7:**
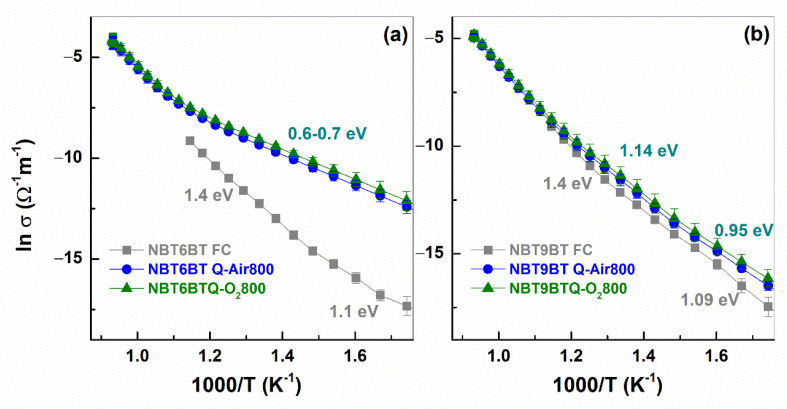
Arrhenius plots of conductivity for the furnace cooled and quenched samples of (**a**) NBT6BT and (**b**) NBT9BT. The plots for the quenched samples correspond to the in situ annealing in air and oxygen atmosphere.

Figure 6 depicts the Nyquist plots of quenched samples measured in situ during the annealing step at 600 °C, in comparison to the furnace cooled specimen. Both quenched NBT6BT and NBT9BT indicate lower resistivity, with stronger decrease noted for quenched NBT6BT. This is in accordance with prior reports [39,40]. From the Arrhenius plots of conductivity (Figure 7), the slope is used to estimate the activation energy (denoted with units eV in the figure). Albeit the increase in bulk conductivity of the quenched samples, the oxygen vacancy concentration is just at the threshold limit for high ionic conductivity [40].

From the above, it is clear that although the increase in conductivity of quenched samples cannot be disputed, the unchanged conductivity in the annealing study hints at additional mechanisms at play. Note that prior works indicate an increase in the lattice distortion upon quenching [18,23,38] and a counter effect upon annealing at 800 °C [38]. Considering that the Bi^3+^ ion at the A-site exhibits off-centered displacements even for the furnace cooled specimen [41,42], it is plausible that the off-centering effects are more pronounced upon quenching. The off-centered displacements enhance further along the polar direction upon quenching [43], thus improving the lattice polarizability. This then reflects as the observed increase in average lattice distortion, corroborated by the recent structural and microstructural investigations on the quenched samples [37,43].

### 3.3. Electromechanical Hardening

**Figure 8:**
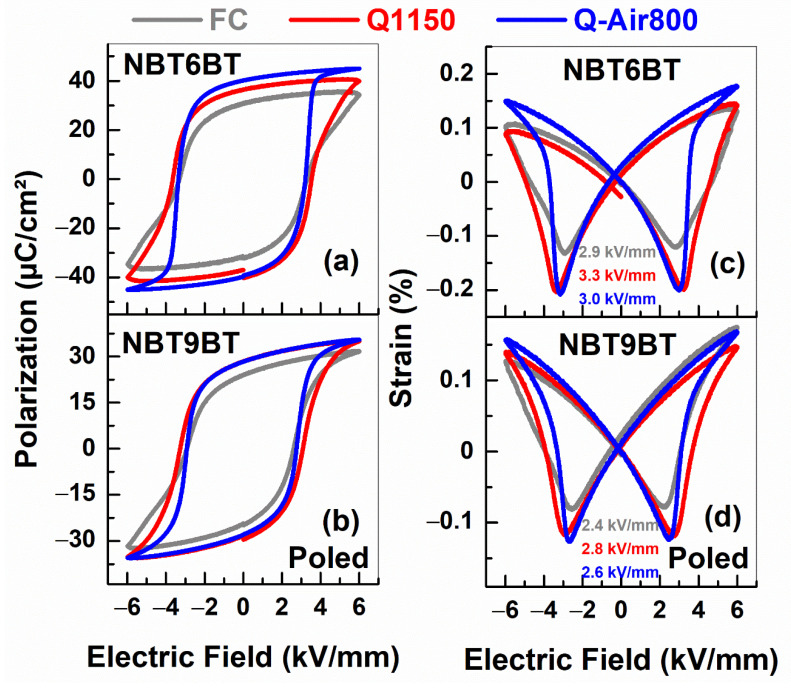
Polarization- and strain-field hysteresis of furnace cooled, quenched and quenched samples subjected to annealing in air for (**a**,**c**) NBT6BT and (**b**,**d**) NBT9BT obtained in the poled state. The numbers with units kV/mm in (**c**) and (**d**) correspond to the coercive fields.

**Figure A1:**
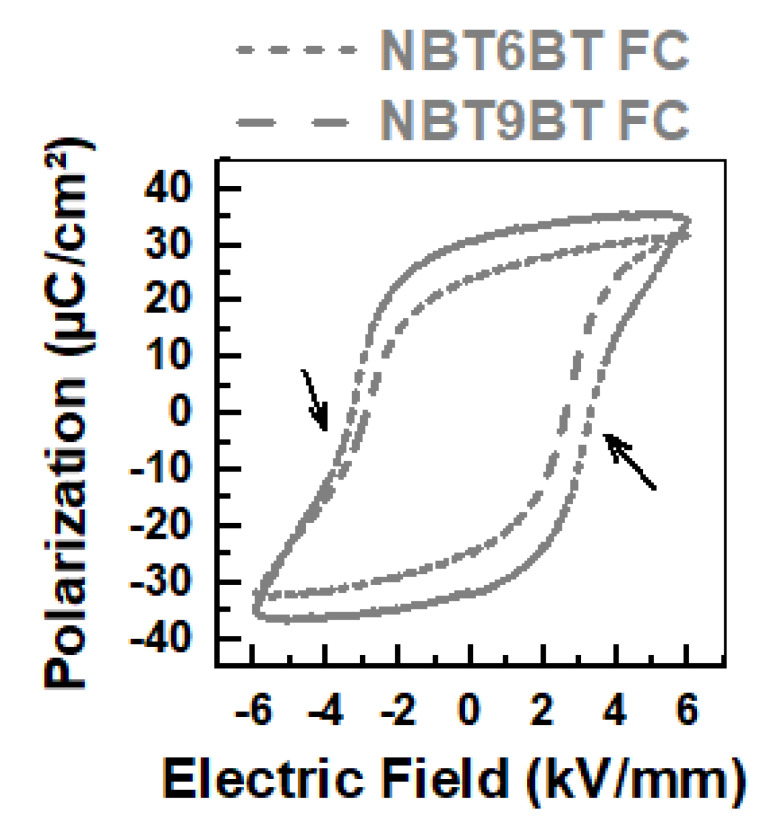
Polarization-field hysteresis of furnace cooled NBT6BT and NBT9BT in the poled state. The arrows denote pinched hysteresis behaviour.

Since the bulk conductivity of the quenched specimen is higher than that of the furnace cooled specimen (Figure 7), the changes in defect chemistry can be expected to alter the electromechanical response. Hardening in ferroelectrics is typically associated with defect dipoles that orient along the direction of spontaneous polarization, that constrain the domain wall motion [31]. However, mobile charged defects can also pin the domain walls in their position and lead to ‘hard-type’ characteristics [32]. Since quenched samples exhibit enhanced conductivity (Figure 7), hinting at increased oxygen vacancy concentration [23,38], the electromechanical properties are investigated to probe into the hardening effects. The characteristic feature of hardened ferroelectrics is the decrease in remanent polarization and strain and increase in coercive field and mechanical quality factor. Since NBT100*x*BT is a non-ergodic relaxor, the hardening behavior is characterized after poling, upon which, the material transforms to the ferroelectric state. Given the similar T_F-R_ (Figure 1g,h and Figure 2g,h, Table 1) and bulk conductivity (Figure 5 and Figure 7) for air/oxygen annealed samples, the electromechanical properties were measured only for quenched samples subjected to annealing in air atmosphere. Upon poling, furnace cooled samples of NBT6BT and NBT9BT exhibit pinched hysteresis response (Figure A1), typical for these materials. However, this is absent for the quenched samples with a 15% increase in the remanent polarization for both NBT6BT Q1150 and NBT9BT Q1150 in comparison to FC samples (Figure 8a,b). NBT6BT Q-Air800 exhibits a further 10% increase in the remanent polarization in comparison with NBT6BT Q1150. A striking feature is the 35% and 18% increase in total strain for NBT6BT Q1150 and NBT9BT Q1150 respectively. Similar to the polarization response, a further 16% and 8% increase in the total strain is observed for NBT6BT Q-Air800 and NBT9BT Q-Air800, respectively, in comparison with Q1150 samples. Note that in the unpoled state, the hysteresis response of furnace cooled and quenched NBT6BT were comparable, while quenched NBT9BT exhibited a difference that was attributed to the spontaneous ferroelectric order that develops in the material [23].

The polarization and strain hysteresis in the poled state exhibits a marked departure from the expected influence of oxygen vacancies constraining the domain wall mobility; instead, the increase in total strain and remanent polarization is plausibly a signature of enhanced ferroelectric order. This is in accordance with recent reports that establish increase in volume fraction of polarized regions [44] and enhanced lamellar domain contrast [37]. The hysteresis response of Q-Air800 samples are in contrast to the depolarization behaviour, wherein the T_F-R_ of Q-Air800 samples are similar to FC (Table 1). The increase in remanent polarization and total strain upon annealing the quenched samples can be rationalized considering the fact that the annealing tends to equilibrate the defect concentration, thus easing the domain wall mobility. The hardening effects, although minor are noted from the increase in coercive field for Q1150 and its decrease upon subsequently annealing the quenched samples in air (Q-Air800). The coercive field (established from the minimum in strain-field hysteresis) is 2.9, 3.3 and 3.0 kV/mm for FC, Q1150, and Q-Air800 of NBT6BT, respectively. The coercive field is 2.4, 2.8 and 2.6 kV/mm for FC, Q1150, and Q-Air800 of NBT9BT, respectively.

**Figure 9:**
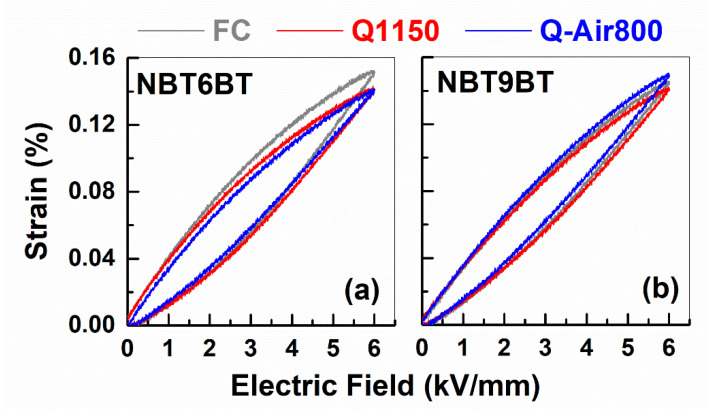
Unipolar strain-field hysteresis of furnace cooled, quenched and quenched samples subjected to annealing in air for (**a**) NBT6BT and (**b**) NBT9BT obtained in the poled state.

**Figure 10:**
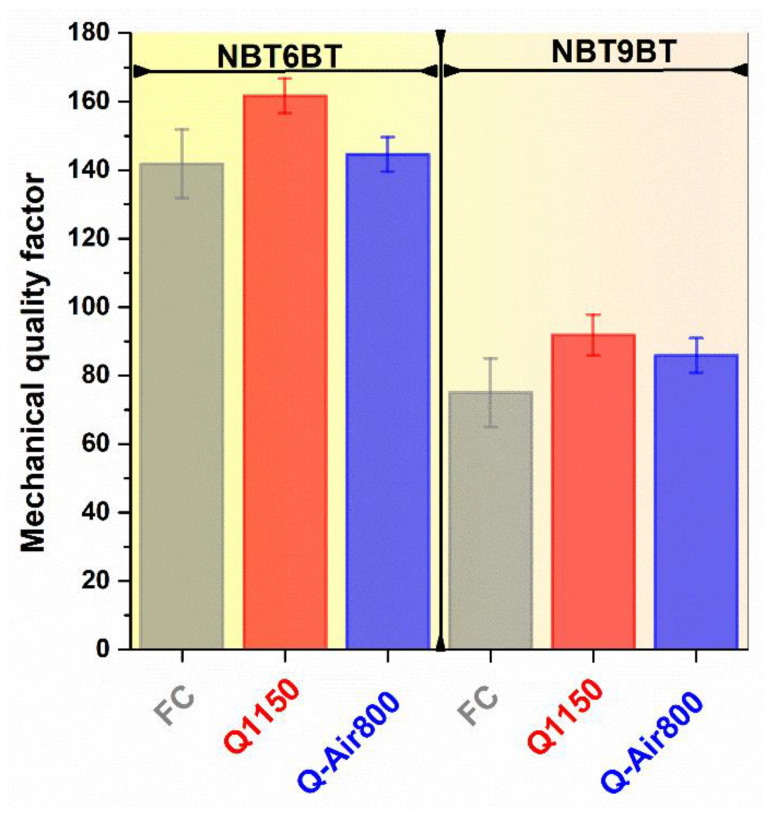
Mechanical quality factor of furnace cooled, quenched and quenched samples subjected to annealing in air for NBT6BT and NBT9BT.

A distinct signature of hardening is established from the unipolar strain-field hysteresis (Figure 9) and mechanical quality factor (Figure 10). Note that the unipolar hysteresis response is almost identical for FC, Q1150, and Q-Air800 samples, indicating similar hysteresis losses.

The mechanical quality factor is a dimensionless measure of the losses of a piezoelectric material at resonance [33] and is one of the figures of merit to gauge materials for high power applications, such as ultrasonic motors, transformers, and high intensity focused ultrasound. The mechanical quality factor exhibits a 10–15% increase upon quenching. Negligible changes in the mechanical quality factor and coupling coefficient upon quenching was also previously noted for pure NBT [18]. These results indicate that the electromechanical hardening effects upon quenching is not as pronounced as is the case with ‘hard-type’ NBT-based materials [12,14,22]. Nevertheless, this makes quenching a promising approach to tailor material properties beyond the limits of chemical doping. For example, 0.5 mole% Zn^2+^ doping in NBT6BT exhibits a T_F-R_ of 143 °C and mechanical quality factor of 287; quenching 0.5 mole% Zn^2+^ doped NBT6BT was demonstrated to increase the T_F-R_ further to 163 °C, while retaining the mechanical quality factor at 280 [14]. Note that, quenching the doped composition resulted in a significant increase in T_F-R_ beyond the upper limit established by doping (with 1 mole% Zn-doping in NBT6BT, T_F-R_ increases only marginally to 150 °C) [14]. A similar observation of increase in T_F-R_ upon quenching, independent of that established by chemical modification was also recently noted for quenching 1 wt.% CuO-added ternary (Bi_1/2_Li_1/2_)TiO_3_-modified NBT6BT solid solution [45].

## 4. Conclusions

Annealing the quenched samples of NBT6BT and NBT9BT leads to a decrease in T_F-R_ but has negligible effects on the bulk conductivity. This indicates no correlation between the defect chemistry and the thermal depolarization of quenched NBT-BT. While quenching does enhance the bulk conductivity, indicating increased oxygen vacancy concentration, it is not significant enough to strongly alter the domain wall mobility and thus results in minor hardening effects, reflected as 10–15% increase in mechanical quality factor. These results provide the premise for combining quenching with the chemical doping strategy to facilitate enhancement of the depolarization temperature without significantly altering the electromechanical properties.

## Figures and Tables

**Table 1:** Characteristic parameters established from the permittivity plots in Figure 1, Figure 2 and Figure 3.

Sample	$\Delta \varepsilon \mathrm{Hz}=(\varepsilon_{100Hz}^{’}$ $-\varepsilon_{1MHz}^{’})\;\mathrm{at}\;40\;{}^{\circ}C\;(\mathrm{Unpoled})$	$\Delta \varepsilon p=|\varepsilon_{unpoled}^{’}$$-\varepsilon_{poled}^{’}|$ at 40 °C and 10kHz	TF-R, °C (Poled)	Tm, °C at 1MHz (Unpoled)	TRE, °C (Unpoled)
**NBT6BT**					
FC	533	656	100	272	231
Q1150	523	486	143	272	259
Q-Air800	664	702	100	261	255
Q-O_2_800	638	680	100	261	250
**NBT9BT**					
FC	657	1041	151	267	268
Q1150	227	825	203	267	283
Q-Air800	549	963	163	259	276
Q-O_2_800	416	966	159	258	272

## Data Availability

Data is contained within the article and Appendix A.
